# Anopheline diversity in urban and peri-urban malaria foci: comparison between alternative traps and seasonal effects in a city in the Western Brazilian Amazon

**DOI:** 10.1186/s12936-022-04274-8

**Published:** 2022-09-06

**Authors:** Anne Caroline Alves Meireles, Lucas Rosendo da Silva, Marlon Ferreira Simplício, Alzemar Alves de Lima, Flávia Geovana Fontineles Rios, Carla Augusta de Menezes, Luiz Henrique Maciel Feitoza, Genimar Rebouças Julião

**Affiliations:** 1 Postgraduate Program in Experimental Biology (PGBIOEXP), Federal University of Rondônia (UNIR), BR-364, Km 9.5, Porto Velho, RO 78900-550 Brazil; 2 Laboratory of Entomology, Oswaldo Cruz Foundation, FIOCRUZ Rondônia, Porto Velho, RO 76812-245 Brazil; 3 INCT-EpiAmO – National Institute of Epidemiology of Western Amazônia, Porto Velho, RO Brazil; 4 Centro de Pesquisa em Medicina Tropical de Rondônia, CEPEM-RO, Porto Velho, RO 76812-329 Brazil; 5 Centro Universitário São Lucas, Porto Velho, RO 76805-846 Brazil

**Keywords:** Human Landing Catch, Seasonality, Barrier screen, Biting behaviour, *An. darlingi*

## Abstract

**Background:**

Continuous vector surveillance and sustainable interventions are mandatory in order to prevent anopheline proliferation (or spread to new areas) and interrupt malaria transmission. Anopheline abundance and richness were evaluated in urban and peri-urban malaria foci at a medium-sized city in the Brazilian Amazon, comparing the protected human landing catch technique (PHLC) and alternative sampling methods over different seasonal periods. Additional information was assessed for female feeding behaviour and faunal composition.

**Methods:**

Anophelines were sampled bimonthly in four urban and peri-urban sites in the city of Porto Velho, state of Rondônia, Brazil. The average number of captured mosquitoes was compared between an PHLC (gold standard), a tent trap (Gazetrap), and a barrier screen by means of generalized linear mixed models (GLMM), which also included season and environment (peri-urban/urban) as predictors.

**Results:**

Overall, 2962 *Anopheles* individuals belonging to 12 species and one complex were caught; *Anopheles darlingi* represented 86% of the individuals. More mosquitoes were captured in the peri-urban setting, and the urban setting was more diverse. The model estimates that significantly more anophelines were collected by PHLC than by the Screen method, and Gazetrap captured fewer individuals. However, the Screen technique yielded more blood-engorged females. The peak hours of biting activity were from 6 to 7 p.m. in urban areas and from 7 to 8 p.m. in peri-urban areas.

**Conclusions:**

Although peri-urban settings presented a greater abundance of anophelines, Shannon and Simpson diversities were higher in urban sites. Each technique proved to be useful, depending on the purpose: PHLC was more effective in capturing the highest anopheline densities, Gazetrap caught the greatest number of species, and the barrier screen technique captured more engorged individuals. There was no seasonal effect on *Anopheles* assemblage structure; however, a more diverse fauna was caught in the transitional season. Biting activity was more intense from 6 to 8 p.m., with a predominance of *An. darlingi*.

**Supplementary Information:**

The online version contains supplementary material available at 10.1186/s12936-022-04274-8.

## Background

Malaria is an infectious disease caused by protozoa of the genus *Plasmodium* [1]. In 2020, about 241 million malaria cases were reported worldwide, with the African continent accounting for 95% of cases [2]. In Brazil, over 145,000 cases were recorded in the same year, 98.7% of which were exclusively in the Amazon region [3]. Five species of *Plasmodium* can cause human malaria; *Plasmodium vivax* and *Plasmodium falciparum* are the main species that burden the public health system in the Brazilian Amazon, where *P. vivax* is responsible for approximately 86% of confirmed cases [2].

Mosquitoes of the *Anopheles* genus are responsible for transmission of *Plasmodium* spp. In Brazil, about 67 species of anopheline mosquitoes have been described, and 49 species have been recorded in the Brazilian Legal Amazon region. *Anopheles albitarsis*, *Anopheles aquasalis*, *Anopheles benarrochi*, *Anopheles braziliensis*, *Anopheles darlingi*, *Anopheles deaneorum*, *Anopheles janconnae*, *Anopheles marajoara*, *Anopheles nunesztovari *sensu lato (s.l.), *Anopheles oswaldoi* s.l., *Anopheles rangeli*, *Anopheles rondoni*, *Anopheles strodei*, *Anopheles triannulatus* s.l., *Anopheles intermedius*, *Anopheles mattogrossensis*, *Anopheles mediopunctatus* and *Anopheles peryassui* have been found naturally infected with malaria parasites in this region [4].

The main malaria vector in northern Brazil is *An. darlingi*, which presents a high anthropophily and can be found feeding both indoors and outdoors, especially during morning and evening twilight hours [4–7]. *Anopheles (Nyssorhynchus) darlingi* is widely distributed in areas of low altitude in South America; this species is absent in the Northeast dry areas of Brazil, in the far South of the country, and in areas of high altitude [5]. Some conditions have challenged malaria elimination programmes such as anthropogenic processes (mining and deforestation), asymptomatic parasite carriers, recurrent *P. vivax* relapses, anti-malarial drug resistance, vector control limitations due to widespread insecticide resistance, in addition to the occurrence of particular epidemiological scenarios including urban, indigenous, or border malaria [8–10]. In urban settings, evidence of local malaria transmission has proven uncommon, and there are many unanswered questions regarding its entomological parameters, such as natural infection rates, identification of *Anopheles* species, and their temporal/spatial distribution [10].

In this regard, several studies have brought to attention the need to resume investments in vector surveillance approaches and sustainable interventions, since the underestimation and weakening of these measures may provide an opportunity for vector population recovery in a given location or even colonization of a new area.

Training and maintaining entomologist teams, community participation, strengthening collaborations, and mixed control strategies must be encouraged; political support will be necessary as well. Many gaps in knowledge still exist in Brazil, and malaria surveillance should be improved with updated mapping of anopheline species, their vectorial competence, molecular taxonomic status, and breeding site records [11, 12]. Hence, vector surveillance is crucial to providing information on species composition, density, behaviour, biology, and natural infection by *Plasmodium* spp. [13]; this tool is the cornerstone to defining control and prevention strategies and investigating the effects of intervention efforts on malaria transmission [10–12].

The monitoring of adult anopheline mosquitoes can be based on entomological collections performed using a series of methods that aim to generate information about the vector mosquito and its relationship with the host [14]. Several techniques employ synthetic and/or physical stimuli in order to capture *Anopheles* spp. mosquitoes, such as BG-Malaria traps [15], BG-Sentinel traps [16], CDC traps (Centers for Disease Control and Prevention®), Shannon tents, screen traps [17, 18], and Mosquito Magnet® traps [19]. Entomological collection using humans as attractive bait is the most frequent and considered the gold standard mainly due to its effectiveness in capturing anthropophilic mosquitoes, which potentially infect humans. However, the Human Landing Catch technique (HLC) involves ethical issues similar to those raised by controlled infection studies in humans, due to the collector’s exposure, as well as the fact that the results are influenced by differences in attractiveness among operators [20]. In this context, alternative practices for capturing these vectors can help reduce the risks to humans [21], for instance tent traps baited with protected human/vertebrates which prevent mosquito bites [22, 23].

Several studies have sought to develop alternative methodologies/devices for vector sampling that take into account behavioural stimuli and biological traits, and specifically, do not constitute a risk to the collector’s health. The main outcomes assessed their efficiency in collecting mosquitoes in different environments and conditions as promising and feasible techniques that can be included in routine surveillance [24–26]. Thus, the present study aimed to compare anopheline diversity (abundance and richness) between protected HLC and alternative sampling techniques in urban and peri-urban malaria foci in a municipality within the Brazilian Amazon—Porto Velho—over different seasonal periods, providing additional information about female feeding behaviour and faunal composition. The municipality of Porto Velho accounted for 54% of the 14,412 autochthonous malaria cases recorded in the state of Rondônia in 2021, according to the federal government's Interactive Malaria Bulletin (https://public.tableau.com/app/profile/mal.ria.brasil#!/). Since urban cores comprise the largest human agglomerations, these populations may be at risk for the disease, and thus the elimination of malaria in these areas is a public health priority, as well as studies on vector species of *Plasmodium* spp.

## Methods

### Study areas

**Fig. 1:**
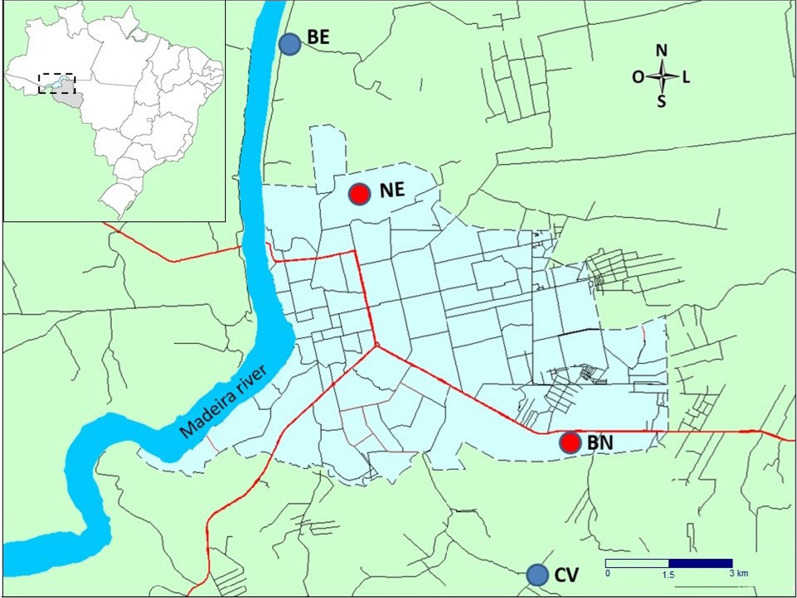
Mosquito sampling sites in the urban and peri-urban settings of Porto Velho—Rondônia, Brazilian Amazon. City of Porto Velho: red circles; urban areas: blue circles; peri-urban areas: blue circles. Image from GPS TrackMaker (Version 13.0.0542)

Sampling was carried out during the months of February, April, June, August, October, and December 2018. Four sampling sites were selected in the city of Porto Velho (Fig. 1), which is the capital of the state of Rondônia, corresponding to the following neighbourhoods/localities: Nova Esperança (NE), Bairro Novo (BN), Belmont (BE), and Colônia Viçosa (CV). Two sites were selected in urban settings (NE = 8°'32.18" S, 63° 52′ 5.96″ W and BN = 8° 48′ 8.74″ S, 63° 48′ 14.28″ W) and two different sites were selected in peri-urban settings (BE = 8° 39′ 30.60″ S, 63° 54′ 37.50″ W and CV = 8° 53′ 10.59″ S, 63° 49′ 55.81″ W).

Briefly, the sites selection criteria were the number of malaria cases, administrative urban boundaries, presence of vegetation and water courses, and anopheline occurrence. Epidemiological data were gathered from the Malaria Epidemiological Surveillance Information System (SIVEP-Malaria), stratified by probable place of infection in the city of Porto Velho, between 2015 and 2017, and neighbourhoods/localities were searched and plotted in the administrative maps of the city of Porto Velho. Thirteen locations with the highest number of malaria cases were listed (Additional file 1: Table S1, Fig. S1). Then, a survey of anophelines was carried out at twenty-five sites in order to verify the presence of *Anopheles* mosquitoes (Additional file 1: Table S2); vegetation cover and watercourses close to residential areas were also taken into account when selecting the four sites. Urban sites were located at the urban core and expansion area and peri-urban sites were peripheral, about 1–4 km from urban administrative boundaries (Additional file 1: Fig. S1); sites were about 5 km apart.

### Sampling techniques

**Fig. 2:**
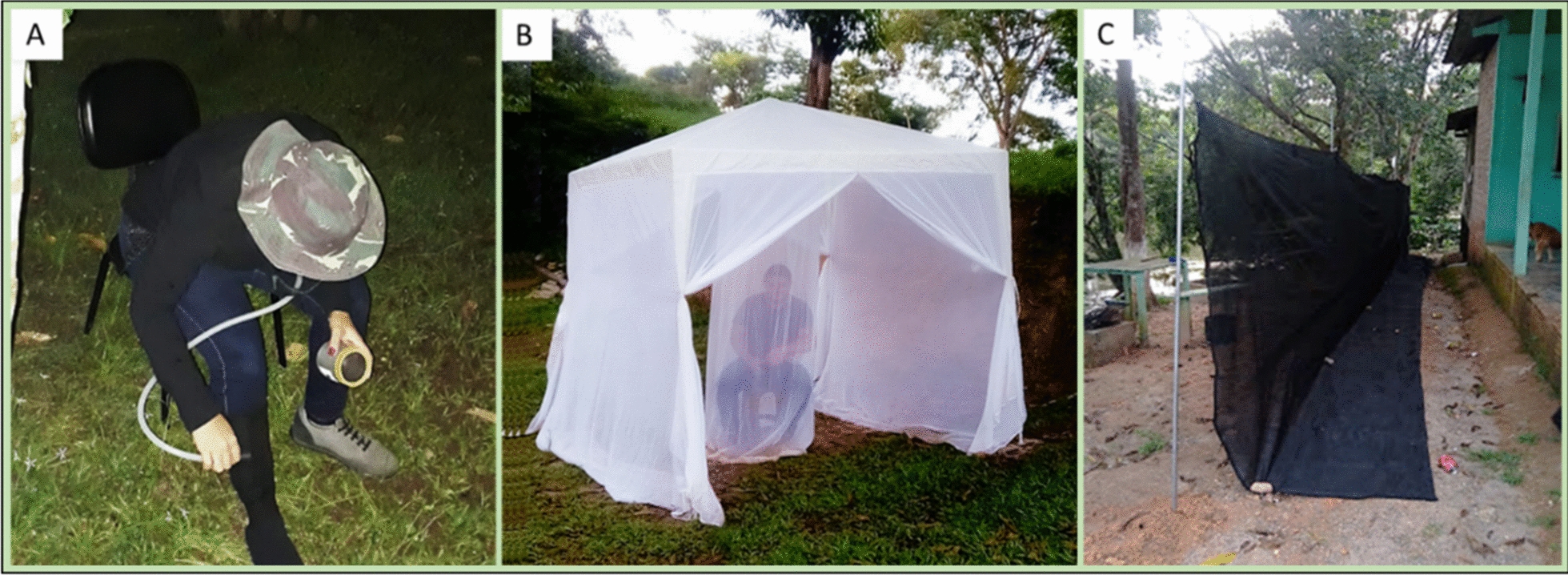
Anopheline capture techniques employed. **A**: Protected Human Landing Catch—PHLC, **B**: Tent trap—Gazetrap, **C**: Barrier Screen—Screen

For adult mosquito capture, three sampling techniques were employed, based on the efficiency of anopheline capture reported in the literature, costs and benefits, logistical feasibility, and availability of materials. The protected human landing catch method—PHLC (Fig. 2a), and the alternative techniques, barrier screen and tent trap (Gazetrap) (Fig. 2b), were performed simultaneously for 6-h/night (from 6 to 12 pm). Techniques were performed exclusively in the outdoors, avoiding extradomiciliary habitat variability such as animal shelters, forest edges, movement of humans, etc., PHLC was considered the "gold standard" for anopheline collection, and it has been employed in several comparative and surveillance studies [27]. Briefly, PHLC consisted of capturing anopheline females that landed on human legs and feet protected by socks, before they started blood-feeding [28], with the aid of a manual suction aspirator and a flashlight with an LED light. PHLC was performed for 45 min every hour and the last 15 min were used for barrier screen sampling. The barrier screen (hereafter, referred to as “Screen”, 15 m width × 2 m height) (Fig. 2c) was built as described in Moreno et al. [17]; however, the Screen colour was changed from green to black. The resting mosquitoes were sampled on both sides of the Screen using a manual suction aspirator for 15 min during every hour of collection. The tent trap (hereafter, referred to as “Gazetrap”) incorporated components and materials based on two tent traps described in the literature. The Gazetrap was built from a gazebo canopy tent (3 × 3 m), and as proposed by Russell et al. [22] to a commercial tent, two sides were closed and two remained open; unlike these authors, only human bait was used. An inner chamber was included based on a Brazilian tent, Mosqtent®, to protect the humans from mosquito bites. Mosqtent® is also a gazebo-like tent, built with an elaborate and intricate double-chamber trap, based on human attractants [23]. After an interval of 45 min every hour, the sides were closed and mosquitoes inside the Gazetrap were collected using manual aspiration and electric suction for 15 min. Sampling techniques were placed from 1 to 30 m from the dwellings, always between the dwelling and the watercourse, which were about 5–90 m apart. Riparian habitats and outdoors were occupied by managed vegetation, with gardens, fruit orchards, and cassava plantations. Except for "Bairro Novo", all locations had free-ranging hens in the outdoor environment.

### Study design

For each site, the anopheline collections were carried out over three consecutive days, bimonthly throughout the year 2018, totalling six sampling events, two during the rainy season (February and December), two during the dry season (June and August), and two events during transitional seasons (April and October). The three sampling techniques were employed simultaneously, with a minimum distance of 20 m between them, at each site. There was an hourly alternance of collectors between techniques in each night-sampling. Overall, 18 nights of sampling efforts were performed for each locality, and total effort of 432 h for Gazetrap, 324 h for PHLC, and 108 h for Screen. Mosquitoes were separated in plastic cages by site, collection hour, and capture technique and transported to the Entomology Laboratory at FIOCRUZ-RO. Mosquitoes were maintained alive and fed with cotton soaked in 10% sucrose solution until they were identified taxonomically. Adult mosquitoes were anesthetized with ethyl acetate PA and specimens were morphologically identified using stereo microscopes and dichotomous keys as proposed by Consoli and Lourenço-de-Oliveira [5] and Forattini [29]. The nomenclature of *Anopheles* mosquitoes followed the classification system proposed by Harbach [30]. After performing taxonomic delimitation, the anopheline species/taxa were counted and stored (by date, site, sampling type, collection time, and blood feeding status) in microtubes at − 20 °C for further molecular detection of *Plasmodium* spp. Whenever possible, two to four individuals of each anopheline species were mounted with an entomological pin to be deposited in the COLRO—the Entomological Collection of Fiocruz Rondônia and INCT-EpiAmO.

### Data analysis

Generalized linear mixed models (GLMM) and generalized linear models (GLM) were used for abundance data analysis (Additional file 2: Appendix S1). Anopheline abundance was considered to be the number of individual mosquitoes recorded hourly for each three-day sampling event. The city setting (urban or peri-urban), technique (PHLC, Screen, or Gazetrap), and season (Rainy, Dry, or Transitional) were set as predictors. Since ecological data of vector counts are usually discrete variables and rarely assume normally distributed errors [31], models were carried out using negative binomial errors and “log” link (; glm.nb function—*MASS* package; nbinom2()—*glmmTMB* package). Due to the hierarchical/nested nature of study design, the variables “Site” (clustered by city setting), sampling “Day”, and “Sampling event” were set as random effects in the models, taking into account probable temporal and spatial autocorrelation in the outcomes. The proportion of estimated variance for model components (random and fixed factors) was examined by comparison of GLMM’ pseudo-R-squared (conditional and marginal R^2^). Model comparisons were carried out based on maximum likelihood, using SBC values (Schwarz's Bayesian criterion) for each fitted model (BIC function/*stats*). The models with the lowest SBC values were considered the best approximation models and compared with the null models. Model adjustment was analysed by means of diagnostic plots of the residual adequacy (normality, homoscedasticity, and outliers). Complementary analyses were performed to examine if the responses obtained on a given sampling day/event were related to previous sampling in time using testTemporalAutocorrelation() function from *DHARMa* package (Additional file 2: Appendix S1). Individual-based rarefaction curves were used to estimate and compare both the absolute species number (species richness) and the *Anopheles* species diversity between levels of predictors (settings, techniques, and seasons). Diversity estimates and its 95% confidence intervals were based on the Hill numbers: richness (q = 0), Shannon diversity (q = 1), and Simpson diversity (q = 2), using a matrix of abundance data for each anopheline species. Further, individual-based rarefaction (interpolation) and extrapolation curves of Hill diversity were plotted with 1000 bootstrap replications and 3500 individuals as endpoint (for graphical purposes), using the package iNEXT [32]. Individual-based rarefaction made it possible to estimate the expected richness in a small subset of “n” individuals drawn from the reference sample (observed abundance of the sampled species) [33]. Differences and similarities in the *Anopheles* species composition were tested by Permutational Multivariate Analysis of Variance (PERMANOVA) and Permutational Analysis of Multivariate Dispersions (PERMDISP). For this, Sørensen distance matrices were computed using presence/absence data and the multivariate analyses evaluate whether the within-group distances were different/similar from the between-group distances, take into account seasons and sampling techniques. Nonmetric multidimensional scaling (NMDS) plots were used to depict *Anopheles* assemblage dissimilarities among predictor levels. The R *vegan*, *lattice*, and *permute* packages were used in the compositional analyses. Mosquito biting behaviour (female engorgement and hourly activity) was graphically inspected in order to evaluate spatial and temporal patterns, and trap efficiency. The model estimate outputs were tabulated using *sjPlot* 2.8.4 and *sjmisc* 2.8.5. All graphs and analyses were performed in the R platform, version 3.6.0.

## Results

Overall, 6136 dipterans were collected, belonging to nine Culicidae genera, biting midges (Ceratopogonidae: *Culicoides* spp.), and sandflies (Psychodidae: Phlebotominae) (Additional file 3: Tables S3 and S4). The genus *Anopheles* was the most abundant; 2962 (49.1%) individuals were identified, including 12 species and one complex (*An. mediopunctatus/costai/forattini*). Seventeen individuals were damaged and remained in the genus level delimitation (Additional file 4: Tables S5, S6, and S7). The statistical modelling approach with fixed (city setting, season, and sampling technique) and random effects (sampling day *per* event) revealed distinct patterns for the abundance estimates. The number of anopheline individuals varied strongly with city setting, sampling technique, and collection day in each sampling event, included as random factor. In turn, anopheline diversity was dependent on the Hill numbers, as result from the relative abundance of mosquito species, and *Anopheles* assemblage composition did not vary between seasons and sampling techniques.

### Effects of city setting, season, and sampling technique on anopheline abundance

**Fig. 3:**
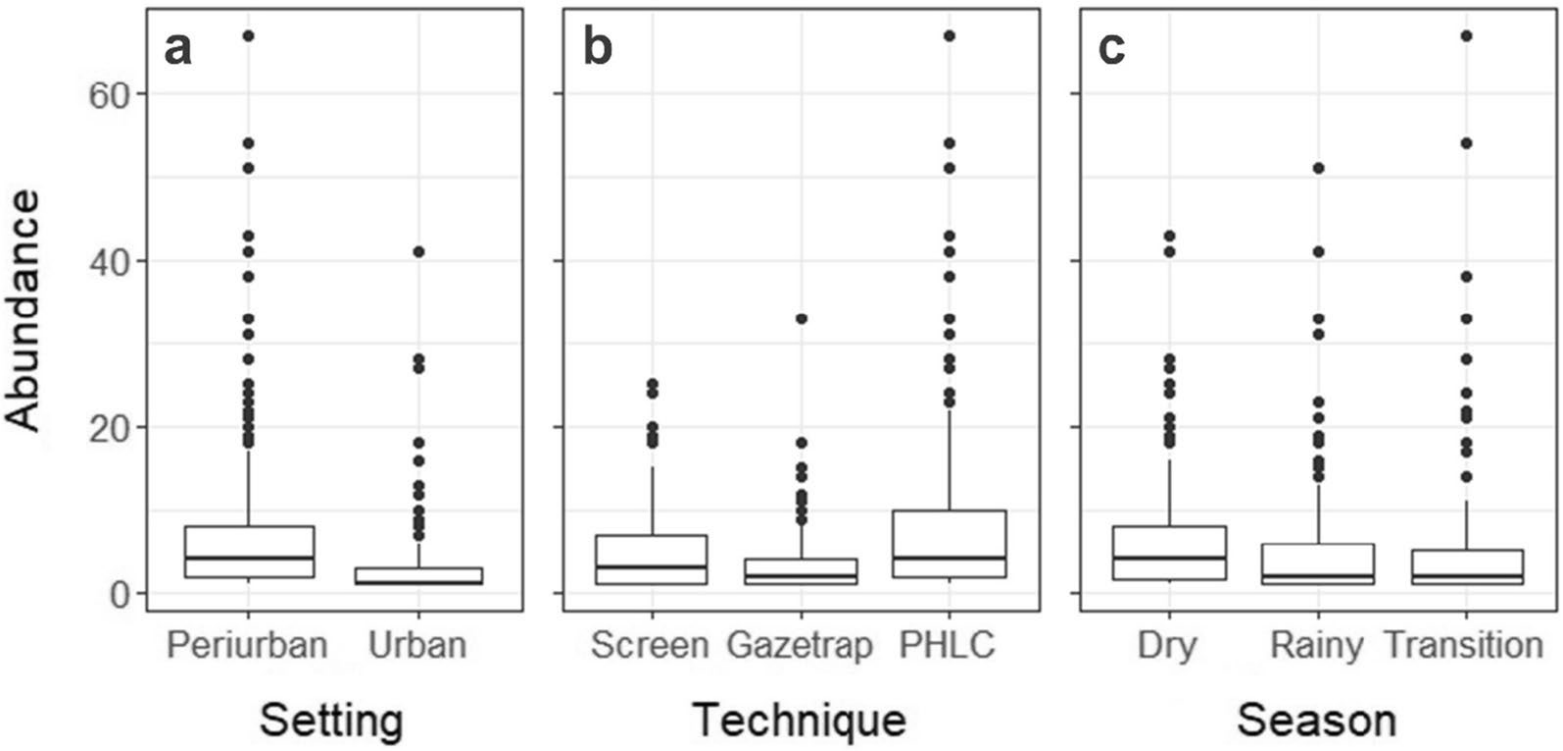
*Anopheles* spp. abundance. Abundance was computed as the number of individual mosquitoes in the urban and peri-urban settings (**A**), captured by different sampling techniques (**B**), during the dry, rainy, and transition seasons (**C**)

More anophelines were captured in the peri-urban sites (Fig. 3a), differing significantly from urban localities (*z* = − 8.61, p < 0.001), and in the months of June and August, during the dry season (Fig. 3c); however, no significant seasonal difference was observed on anopheline abundance (*z* = − 1.18; − 1.05; p > 0.05) when model was adjusted for collection day in each sampling event (see next section). The average number of *Anopheles* individuals also varied significantly among the three sampling techniques. As expected, PHLC was the most productive technique, capturing 1,662 anophelines (Fig. 3b) and anopheline abundance estimates for PHLC significantly differed from Gazetrap and Screen (*z* = 5.61, p < 0.001). There was no variation between estimates for Gazetrap and Screen in the fixed- and random-effects model (Additional file 4: Table S8).

### Temporal patterns in the abundance of *Anopheles* spp.

The best-fit model suggested no seasonal variation in the anopheline abundance when considering collection day *per* sampling event as a random effect, since consecutive daily samplings were considered the main source of serial dependence. Although there is no significant temporal autocorrelation (Additional file 2: Appendix S1), collection day explained part of variance in the model. The variance in the mean number of *Anopheles* spp. individuals was mainly due to between-group differences (τ_00_ = 0.70), i.e., between the bimonthly sampling events (Additional file 4: Table S8). Besides, random effect accounted for about half of the variance computed for the entire model (Conditional r-squared, R^2^c = 0.067) since pseudo-R^2^ estimated for fixed predictors was 0.031 (Marginal r-squared, R^2^m, Additional file 4: Table S8).

### Effects of city setting, season, and sampling technique on anopheline diversity

**Fig. 4:**
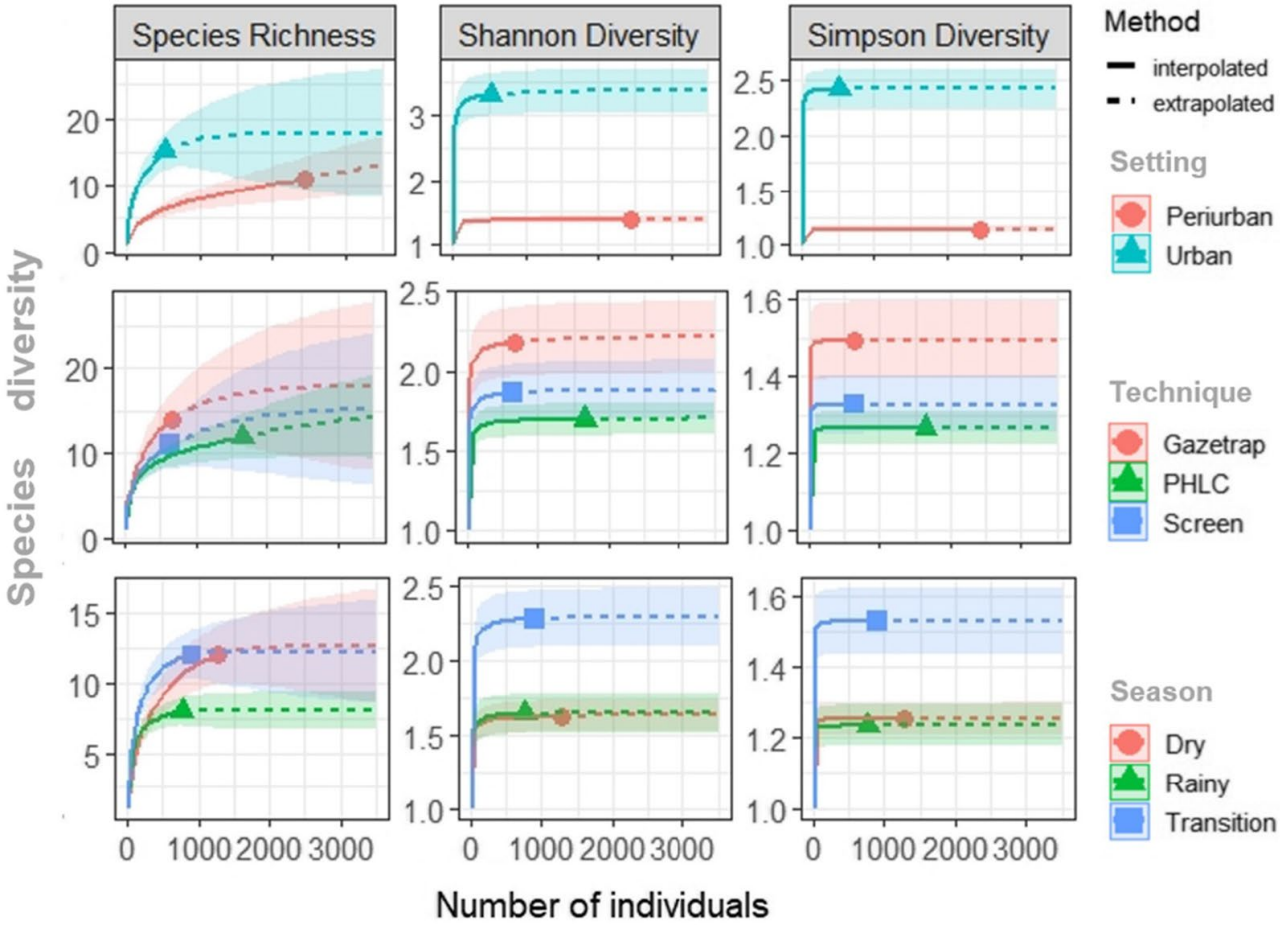
Individual-based rarefaction (solid lines) and extrapolation curves (dashed lines) of *Anopheles* diversity from Porto Velho, in the Brazilian Amazon. Diversity based on Hill numbers (0, 1, 2) were estimated for city settings, sampling techniques, and seasons. Geometric shapes in the lines depict the reference sample for each comparison level

The estimates for absolute number of anopheline species (Richness, q = 0) did not differ between urban and peri-urban settings; PHLC, Gazetrap, and Screen techniques; and Dry and Transitional seasons. There was a partial overlap in the 95% confidence, and a tendency of the lowest number of *Anopheles* species was observed in the Rainy season. However, the estimates of Shannon and Simpson diversity indices showed different patterns: urban setting, Gazetrap technique, and Transitional season presented a more diverse fauna of *Anopheles* species (Fig. 4).

### Proportion of *Anopheles* individuals per setting × technique and technique × hours

**Fig. 5:**
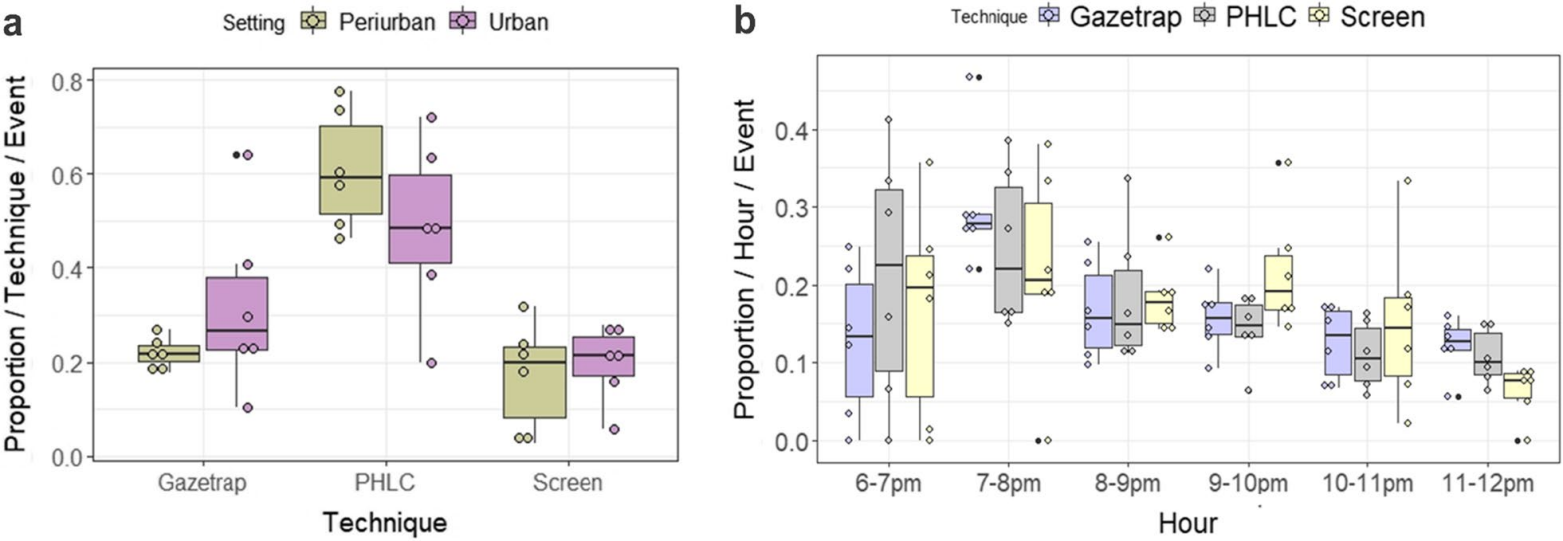
Percentage of individuals captured by **a** each of the three sampling techniques in urban and peri-urban settings, and **b** by sampling techniques and collection hours

All techniques showed similar performance when comparing the proportion of specimens captured in urban versus peri-urban settings (Fig. 5a). Although the alternative Screen and Gazetrap methods captured fewer anophelines, there was a proportionality between PHLC and these techniques in terms of the number of individuals collected over the sampling hours (Fig. 5b).

### Anopheles biting activity

**Fig. 6:**
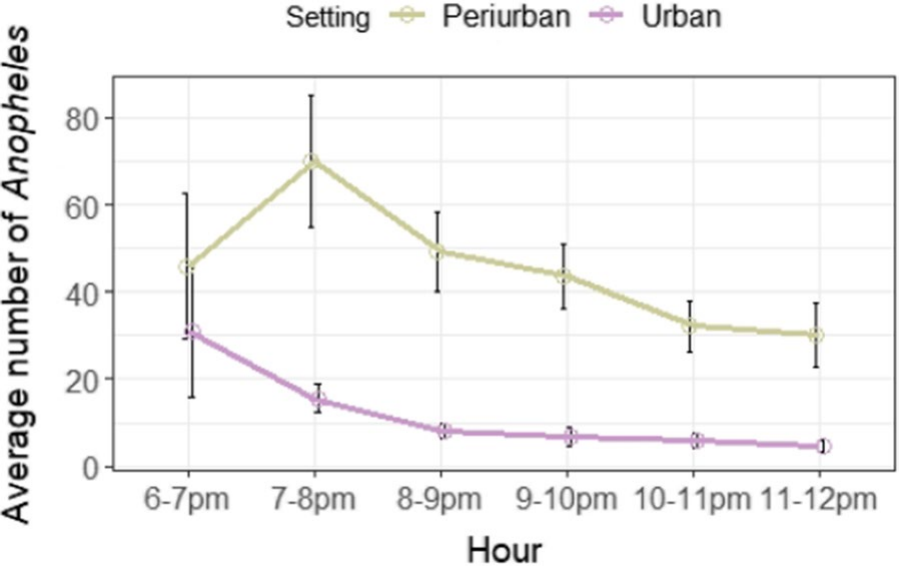
Average number of *Anopheles* spp. individuals caught *per* hour in the urban and peri-urban settings

In general, anophelines were more frequently captured between 6 and 8 p.m., but there was a clear difference in the biting activity of anopheline mosquitoes between urban and peri-urban settings (Fig. 6). The first sampling hour (from 6 to 7 p.m.) was the main peak of activity in the urban sites (n = 185), when the highest number of *Anopheles* specimens were caught. However, anopheline catches were higher during the second hour (from 7 to 8 p.m.) in peri-urban areas, with an average slightly higher than in the urban sites ($\overline{X }$= 5.4 and $\overline{X }$= 5.7). After 8 p.m., both the absolute and average number of individuals decreased. Anopheline richness did not show clear patterns between the collection periods (Fig. 6).

### Feeding status

**Fig. 7:**
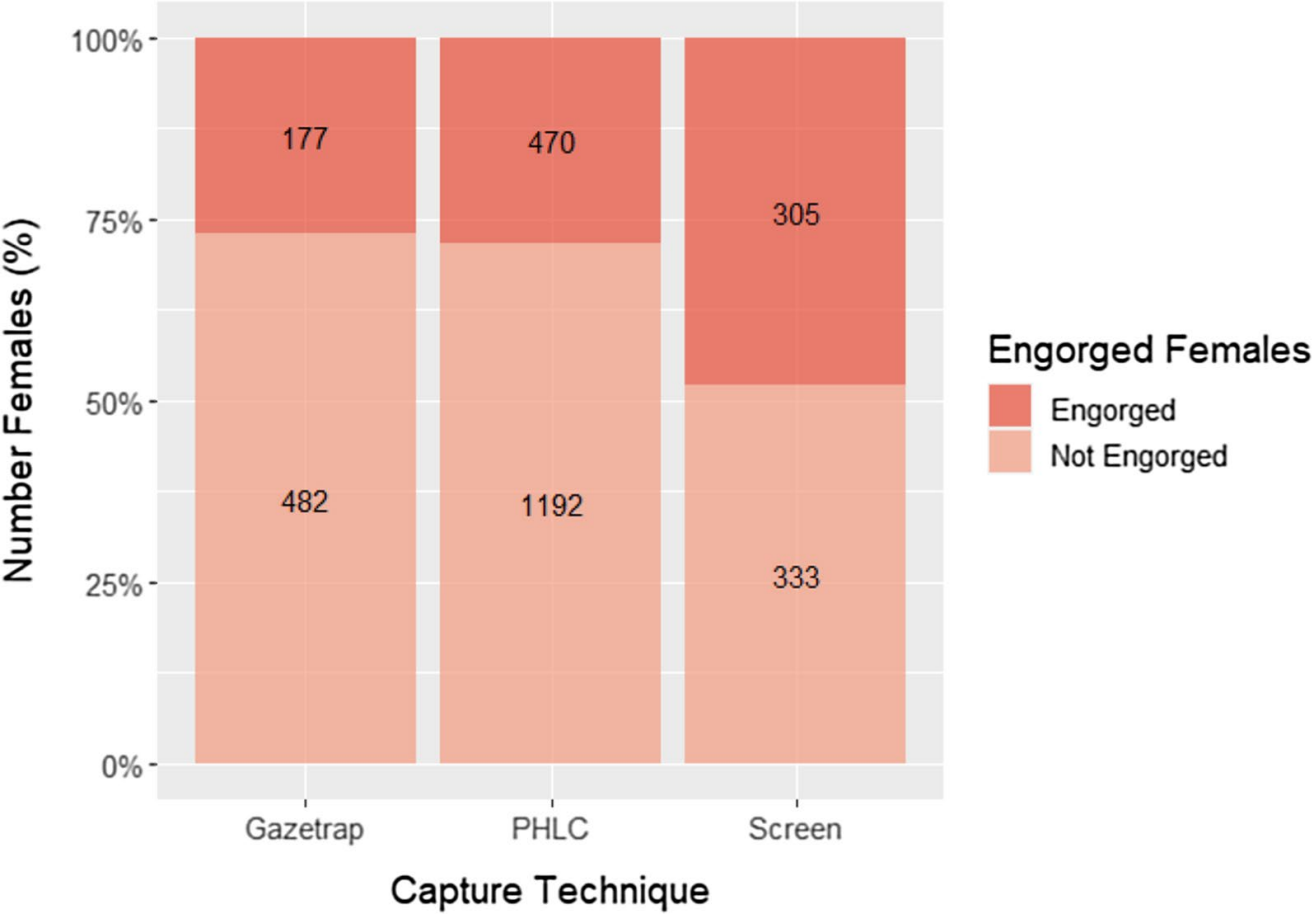
Proportion of engorged anophelines according to sampling technique

Almost all of the mosquitoes captured were females (n = 2959), and about a third (33%, n = 952) were engorged with blood upon capture. The proportion of engorged and non-engorged females varied among the sampling techniques. As expected, more engorged mosquitoes (47.8%, n = 305) were captured relatively in the Screen than in PHLC (28.3%, n = 470) or Gazetrap (26.9%, n = 177) (Fig. 7).

### Anopheles species composition

**Fig. 8:**
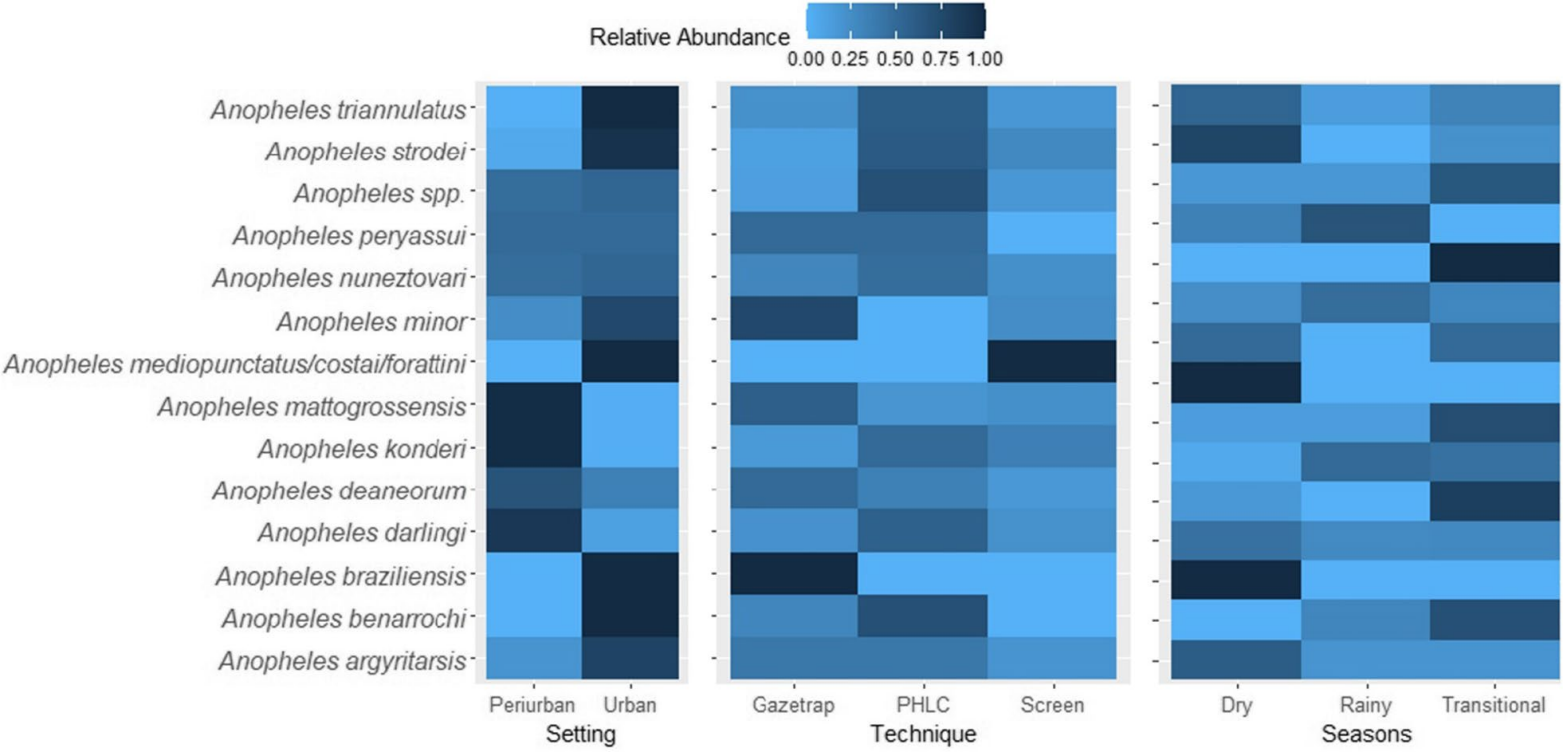
Relative abundance of *Anopheles* species by city setting, sampling technique, and season. Relative abundance was determined for each species and compared between levels of the variables Setting, Technique, and Season. Values range from 0 to 1, with relative abundances close to 0 in light blue, and close to 1, dark blue in the colour gradient

*Anopheles darlingi* was the predominant species in all sites and accounted for about 86% of the total individuals captured (n = 2563), followed by *An. triannulatus* (5.98%, n = 177), *An. mattogrossensis* (3.24%, n = 96), and *An. konderi* (1.49%, n = 44). *An. strodei*, *An. nuneztovari*, *An. benarrochi*, *An. minor*, *An. argyritarsis*, *An. deaneorum*, *An. mediopunctatus/costai/forattini*, *An. braziliensis* and *An. peryassui* were represented by less than 1% of the individuals. The species *An. benarrochi*, *An. braziliensis* and the *An. mediopunctatus/costai/forattini* complex were found only in urban sites (Fig. 8). In addition, the alternative techniques were exclusively responsible for their captures. *Anopheles braziliensis* was captured only in Gazetrap while *Anopheles mediopunctatus/costai/forattini* was unique to the Screen technique (Fig. 8). There were no significant patterns in the *Anopheles* assemblage structure when season and sampling technique levels were compared by PERMANOVA (season: pseudo-F_2,15_ = 1.114, P = 0.382; technique: pseudo-F_2,9_ = 1.173, P = 0.985) and PERMDISP (season: pseudo-F_2,15_ = 1.114, P = 0.382; technique: pseudo-F_2,9_ = 0.166, P = 0.834) tests, visually confirmed by NMDS ordination plots (Additional file 4: Table S9, Fig. S2a,b).

## Discussion

Results of the present study demonstrated that the surroundings and urban core of a city in the Brazilian Amazon may harbour a variety of anopheline species, potential vectors of *Plasmodium* spp., which in turn coexist in both urban and peri-urban environments. This faunal diversity can vary according to location, season, and behavioural aspects of each species, depending on sampling procedures.

### Abundance and diversity of anophelines in the urban-peri-urban setting

Anopheline abundance was the main variable affected by model predictors. The greatest number of individuals were collected in peri-urban areas, a result commonly found in other studies throughout the Amazonian region, mainly in areas with vegetation and newly anthropized areas near watercourses, like in the present study [34, 80]. Despite the low density of anophelines in the urban settings sampled in this study, a high endemicity of malaria is possible even in a low density of vectors [6]. As observed in Manaus—Amazonas state, the expansion and creation of new residential areas, such as neighbourhoods and squatters, preceded malaria outbreaks in some areas of the city [35].

On the other hand, the richness of *Anopheles* spp. did not vary significantly among the Porto Velho sites, though the absolute number of species found in urban areas was higher than in peri-urban areas. This can be explained by the variation in species composition, seasonality, and habits of the anopheline fauna, which can adapt to environments with recent anthropic changes [36]. Surprisingly, individual-based rarefaction/extrapolation estimates revealed a more diverse fauna in urban settings, and these findings contrast with some recent studies about the anthropic impact on *Plasmodium* spp. vectors [37, 38], requiring further investigation, especially those on insect dispersal between urban, peri-urban, and rural areas.

### Abundance and diversity of anophelines and seasons

Malaria vectors can also easily adjust to natural environmental changes, differing in terms of regional, local and species reproductive patterns. An example is the increased density of anophelines during the rainy season due to the creation of temporary breeding sites [39]. However, in urban and peri-urban settings of Porto Velho, more individuals were collected during the dry season, although no statistical significance. This pattern differs from some findings in the region that show an increase in anophelic density during the rainy season and a decrease at the beginning of the dry season [34, 40]. One explanation for this divergence is that in anthropized environments, similar to the city of Porto Velho, the availability of artificial breeding sites may continue to exist, regardless of the season. Thus, the existence and maintenance of these larval habitats can reduce the impact of seasons on the dynamics of vector populations [41–43]. For instance, *An. darlingi* larvae have been found in dams, fishponds, clay pits, containers, and artificial ponds; *An. triannulatus*, in fishponds, artificial ponds, and drum/tanks; *An. argyritarsis*, in drum/tank, plant pots, water tanks, car parts, plastic containers, cans, dams, fishponds, clay pits, artificial ponds, and water tank. *An. nuneztovari*, *An. peryassui*, *An. braziliensis*, and *An. deaneorum* larvae were also collected in dams, fishponds, clay pits, and plastic bottle traps [41, 44–49]. Individual-based rarefaction curves indicated that the transitional season accumulated the greatest diversity of anopheline species. Such pattern was previously described for *Anopheles* and other mosquitoes, and at the Brazilian Amazon, can be also associated with hydrological cycles, flooding, and climatic/environmental changes [50, 51].

### Abundance and diversity of anophelines and techniques

A study conducted in Rondônia in 1999 compared HLC, PHLC, and two types of Shannon trap, and found that about half of the total anophelines were captured with HLC, followed by PHLC (a quarter), and to a lesser extent in Shannon traps [27]. As expected, the presented results confirm the efficiency of the Human Landing Catch technique in assessing anopheline densities at different places and times. For instance, the HLC (and PHLC) technique has previously demonstrated a higher yield in comparison with other traps [25, 52], which can be explained by the fact that the main malaria vector species are quite anthropophilic [25]. Entomological findings also demonstrated the importance of alternative and complementary traps to caught zoophilic species in urban areas, as *An. brasiliensis*, *An. mediopunctatus/costai/forattini*, and *An. minor* caught only Gazetrap tent and Barrier Screen.

Despite PHLC’s superior performance, the data also showed a similar proportion among techniques when comparing the number of individuals collected in each type of city setting and during different sampling hours. Alternative methods such as tent traps and mosquito interception traps have been proven to be feasible and effective for the surveillance of *Anopheles* mosquitoes, both compared to HLC/PHLC and used individually [17, 22, 23]. This variation in the efficiency of collection methods can be influenced by landscape characteristics, anopheline species, and aspects of the local human population, such as their culture and behaviour, among other factors [27]. The rarefaction analysis showed that the alternative techniques achieve higher anopheline diversity than PHLC, and together with the possibility of minimizing such challenges, make these techniques desirable in inventory and biodiversity studies. The individual protection measures can be maintained while performing alternative techniques, and the choice of a particular/single strategy should consider the research/surveillance objectives, and whether there are predefined anopheline species as the target of the study. Also, ethical issue attendance, genus specificity, non-powered components, low technical expertise, logistical feasibility, material availability, mimicry of synanthropic habitats, and high rates of engorged mosquitoes (and those looking for blood sources) were the main benefits associated with the use of these alternative traps, Barrier screen and Gazetrap.

### Feeding status

A higher percentage of engorged individuals were obtained with the Screen method than with the other two capture techniques, a similar result to that recorded in recent studies, especially with anophelines [17, 18, 53, 54]. The positioning of the barrier screen between feeding sites and oviposition and/or resting sites allows for the collection of mosquitoes that were blood-fed or those that were in search of blood meals [53]. These results indicate that properly positioned interception traps can be useful in capturing blood-fed zoophilic, anthropophilic, and opportunistic *Anopheles* mosquitoes. Barrier screen traps have provided important insights about vector species and possible vectors, their flight and feeding behaviour, preferred hosts, and host-seeking periods and peaks [18, 54]. In addition, screen traps can complement PHLC in places with a high density of mosquitoes, reducing collection time and sampling efforts, making it a very economical collection strategy that can be used in remote locations [18], keeping in mind individual protection measures, target species, and malaria surveillance and research goals. For instance, *An. darlingi* was predominantly caught in PHLC.

### Anopheles biting activity

Anopheline biting activity is one of the biological parameters directly related to the malaria epidemiology [55]. In northern Brazil, *Anopheles* mosquitoes show peaks of biting activity at dusk and dawn [56, 57]. Following the same pattern, most urban anophelines in Porto Velho showed their greatest activity between 6:00 p.m. and 8:00 p.m., corroborating other studies carried out in the Brazilian Amazon [39, 55, 58].

Furthermore, it is not uncommon to find variation in peak hours of biting activity, depending on the anopheline species and the place of collection [58–60]. An example of this variation is mosquitoes of the species *An. darlingi*, which have non-twilight habits (9:00 p.m. to 12:00 a.m.); however, they have been found biting during all hours of the night, with peaks occurring at different times [60–63]. Hence, host-seeking and biting activity of some species of anophelines may depend on several factors, since these mosquitoes are very sensitive to variations and anthropic actions, such as deforestation and urbanization, which generate changes in environmental and meteorological conditions (temperature and relative humidity), affecting the availability of suitable sites for vector reproduction. Biting behaviour can also vary according to area and/or geographic location [29].

The review by Stone & Cross [64] points out that environmental changes can exert selective pressure on the feeding behaviour of mosquitoes, which can result in differentiated patterns of malaria transmission. Other determining factors for variations in biting frequency include the season, precipitation, genetic variability, control measures adopted in the location, habits of the local population, quality and variety of hosts, and the dispersion and relative abundance of species [64, 65].

### Anopheles species composition

The twelve species and one anopheline complex found in the city of Porto Velho have also been described in other studies carried out in the Brazilian Amazon, though surprisingly, the high diversity at the urban setting was previously described only for rural areas [34, 39, 56, 63, 66–68]. Despite this, PERMANOVA and PERMDISP tests showed no variance in the *Anopheles* assemblage structure between seasons and sampling techniques, probably due to the restricted number of sampled sites.

*Anopheles mattogrossensis*, *An. konderi, An. strodei*, *An. nuneztovari*, *An. benarrochi*, *An. minor*, *An. argyritarsis*, *An. deaneorum*, *An. mediopunctatus/costai/forattini*, *An. braziliensis*, and *An. peryassui* presented low densities in the sampled urban sites, and are commonly found in other places in the Amazon region [37, 40, 56, 63, 66–70], which can be explained by host preference and the collection techniques used in this study [66], in addition to environmental and seasonal factors.

Regarding epidemiological importance, while *An. darlingi* is the main vector of *Plasmodium* spp. in the northern region of Brazil [5], the species *An. triannulatus* is considered a secondary vector [71], in addition to other species like *An. mattogrossensis*, *An. strodei*, *An. nuneztovari*, *An. deaneorum*, *An. mediopunctatus/costai/forattini*, *An. braziliensis,* and *An. peryassui*, which have previously been found to be naturally infected with human *Plasmodium* [4].

*Anopheles darlingi* was the most abundant species in urban and peri-urban settings, and this predominance over the other species was expected [34, 58, 63, 67–69]. Some studies indicate that *An. darlingi* may be the main vector responsible for the maintenance of malaria in human populations and that a reduction in the population density of this species in a given region can bring about the reduction or even the disappearance of malaria [6, 72, 73]. Several factors may contribute to the vectorial capacity of *An. darlingi*: the species being highly susceptible to *Plasmodium* species that infect humans, anthropophilic habits, its ability to transmit malaria indoors and outdoors, and biting behaviour related to anthropic changes [5, 72, 74, 75].

However, due to their dependence on the physiognomy of the landscape, other anopheline species may show dominance [55]. In some urban sites in Porto Velho, *An. triannulatus* presented similar density and frequency values to those of *An. darlingi*; this co-occurrence was previously described [40, 58, 76]. *Anopheles triannulatus* has an opportunistic and generalist behaviour, benefiting from host availability, being able to colonize altered or anthropized environments, and often becoming more abundant or even dominant over other species [39, 79]. This behaviour, together with the fact that it was found to be naturally infected by *P. vivax*, *P. falciparum*, and *Plasmodium malariae*, ranked this species as an occasional vector of malaria [4, 71].

The unexpected diversity of anopheline mosquitoes in the urban and peri-urban settings may reflect environmental changes due to disorderly urban growth and implementation of hydroelectric power plants near Porto Velho. Its recent urban growth has triggered social and environmental conditions that have enabled the persistence of urban malaria foci in some neighbourhoods of Porto Velho, mainly in peri-urban settings [77]. On the other hand, a hydroelectric artificial lake can provide larval habitats for these mosquitoes. The reproduction and survival of malaria-transmitting mosquitoes, especially *An. darlingi*, depends on the existence of favourable environments with watercourses, vegetation cover, and dwellings close to breeding sites [34, 68, 78], criteria utilized when selecting the sampled sites.

These findings indicate that, in addition to anophelic density and sampling techniques, studies on species taxonomy should be encouraged due to the possible participation of other species considered secondary in the dynamics of malaria transmission [42, 55]. Besides the investigation of infection rates by *Plasmodium* spp. And seasonal density, it is suggested that occasional zoophilic and anthropophilic anopheline species be analysed for their food sources, even in urbanized areas, and that entomological surveillance should be used continuously in these areas in order to strengthen control strategies.

## Study limitations

There are still some potential limitations in the work, such as a small sample size to evaluate spatial patterns (only four sites) in urban/peri-urban areas and the trap study design. Due to their dimensions, Screen and Gazetrap remained in a fixed location throughout the study, which could compromise the outcome randomness (influence of habitat and microclimatic heterogeneity). Also, a minimal trap-distance of 20 m could result in interference/competition between techniques.

## Conclusions

This study highlights the elevated number of anopheline mosquito species in urban settings of an Amazonian city, most of which are *Plasmodium* vectors. Furthermore, the transitional season periods can also present a high diversity of anophelines. Most of the anophelines were captured using the “gold standard” PHLC technique, and the findings suggest that the alternative techniques, Gazetrap and Screen, can complement PHLC, and depending on the purpose, they may be preferable. Non-anopheline mosquitoes, sandflies, and biting midges were also collected, and further studies should evaluate alternative trap efficiency for an integrated surveillance system of other VBD. More studies on mosquito species diversity and taxonomy should be encouraged due to the possible participation of secondary vector species in urban malaria outbreaks.

## Supplementary Information

**Additional file 1. **Site Selection Criteria. **Table S1**. Number of malaria cases in the urban and peri-urban localities from the city of Porto Velho, between 2015-2017. **Figure S1**. Location of 25 sites sampled for anopheline presence in Porto Velho, Rondônia, Brazil. **Table S2**. Details on 25 sites where anopheline presence was recorded, and selected sites in bold.

**Additional file 2. **Residual Diagnostics, Model Validation, and Temporal Autocorrelation Analyses - Anopheline Abundance Data.

**Additional file 3.** Non-anopheline mosquitoes and other Dipterans. **Table S3**. Number of non-anopheline mosquitoes, biting midges, and sand flies caught with Gazetrap, PHLC, and Screen, at urban and peri-urban settings. **Table S4**. Number of non-anopheline mosquitoes, biting midges, and sand flies caught during seasonal periods, at urban and peri-urban settings.

**Additional file 4. **Supplementary results for the number of individuals/species of Anopheles. Model Output – Best-fitted models. PERMANOVA / PERMDISP estimates and NMDS plots. **Table S5**. Anopheles species and individuals (N) captured at urban and peri-urban settings of Porto Velho, state of Rondônia, Brazilian Amazon. **Table S6**. Anopheles species and individuals (N) captured through Gazetrap, PHLC, and Barrier Screen in Porto Velho, state of Rondônia, Brazilian Amazon. **Table S7**. Anopheles species and individuals (N) captured in the Dry, Rainy, and Transitional seasons, at Porto Velho, state of Rondônia, Brazilian Amazon. **Table S8**. Summary of estimates from the best approximation models for anopheline abundance and richness. **Table S9**. PERMANOVA and PERMDISP analyses for Anopheles assemblage structure between seasons and sampling techniques. **Fig. S2**. Non-metric multidimensional scaling (NMDS) plots for graphical visualization of the PERMANOVA and PERMDISP analyses of Anopheles assemblage structure.

## Data Availability

The datasets used and/or analysed during the current study are available from the corresponding author upon reasonable request.

## Abbreviations

PHLC: Protected Human Landing Catch
HLC: Human Landing Catch
GLM: Generalized Linear Model
CDC: Centers for Disease Control and Prevention®
SIVEP-Malaria: Malaria Epidemiological Surveillance Information System
FIOCRUZ-RO: Fundação Oswaldo Cruz Rondônia
INCT-EpiAmO: National Institute of Epidemiology of the Western Amazon
