# Supplementary material for: Anopheline diversity in urban and peri-urban malaria foci: comparison between alternative traps and seasonal effects in a city in the Western Brazilian Amazon
Source: Malar J. 2022 Sep 6;21:258. doi: 10.1186/s12936-022-04274-8 (PMC9450372; doi:10.1186/s12936-022-04274-8)
Supplement: Supplementary file 1 — Additional file 1. Site Selection Criteria. Table S1. Number of malaria cases in the urban and peri-urban localities from the city of Porto Velho, between 2015-2017. Figure S1. Location of 25 sites sampled for anopheline presence in Porto Velho, Rondônia, Brazil. Table S2. Details on 25 sites where anopheline presence was recorded, and selected sites in bold. [file 12936_2022_4274_MOESM1_ESM.pdf]

Additional File 1

Site Selection Criteria

The starting point for site selection criteria was the number of malaria cases in the localities from the city of Porto Velho, between 2015-2017. Localities with no malaria cases were excluded from site selection. Epidemiological data were extracted from the Brazilian National Malaria Surveillance System (SIVEP-Malaria), clustered as "Porto Velho", and stratified by probable place of infection. This stratification was performed for each year separately, and the locations were ranked according to malaria cases. The 30 locations with the highest number of cases were compared between the years 2015, 2016 to 2017 (Table S1).

In order to restrict sampling sites to areas administratively defined as "urban areas", we check each site location on the administrative map of the city of Porto Velho [SEMPOG 2022]. Urban sites were located in the urban core and expansion area and peri-urban sites were peripheral, about 1-4 km from urban administrative boundaries (Figure S1). Urban expansion area was administratively defined as a residential land use of low human density. Urban e peri-urban locations that remained in the rank in these three years were visited (1 night, from 6 pm to 10 pm) in months with highest *Anopheles* density in urban areas (from April to August 2017), and the presence/absence of anophelines was recorded (Table S2). Vegetation cover and watercourses close to residential areas were also taken into account when selecting the four sites.

Table S1. Number of malaria cases in the urban and peri-urban localities from the city of Porto Velho, between 2015-2017.

**Malaria Epidemiological Surveillance Information System - SIVEP MALARIA**<br>Malaria Epidemiological Summary						
**Source Data:** Probable place of infection **State:** RO **County:**PORTO VELHO<br>**Period:** 01/01/2015 a 31/12/2017						
**Year**	**Cod. Locality**	**Locality**	**Class**	**Population**	**Positives**	**IPA**
2015	739	São João	Ranch	126	141	1119
2015	155	Areia Branca - Area Rural (Balneário)	Balneary	270	128	474.1
**2015**	**396**	**Belmont**	**Ranch**	**585**	**98**	**167.5**
2015	121	Bacia Leiteira	Ranch	180	91	505.6
2015	898	Vila do DNIT (8ª Região)	Village	4684	91	19.4
2015	53	Areia Branca	Neighborhood	1647	84	51
2015	140	Km 14	Ranch	247	84	340.1
2015	737	Km13/Santa Helena	Farm	378	79	209
**2015**	**148**	**Colônia Viçosa**	**Colony**	**391**	**60**	**153.5**
2015	141	Uberaba	Ranch	167	52	311.4
2015	44	Triângulo	Neighborhood	3819	50	13.1
2015	407	Maravilha (Rio Madeira)	Ranch	185	48	259.5
2015	630	Novo Engenho Velho	Thorp	144	47	326.4
2015	337	Vista Alegre do Abunã	Village	3973	46	11.6
2015	152	Colônia Japonesa	Colony	98	45	459.2
2015	320	Marmelo - Br 364 Pvh/Acre	Ranch	58	44	758.6
2015	428	Gleba Jamary	Road	280	43	153.6
**2015**	**6**	**Nova Esperança**	**Neighborhood**	**11730**	**42**	**3.6**

2015	151	Jerusalem da Amazonia	Ranch	120	41	341.7
2015	93	Estrada da Penitencia (P.A. Aliança)	Settlement	241	39	161.8
2015	35	Nacional	Neighborhood	8043	38	4.7
2015	99	Terra Santa	Farm	328	35	106.7
2015	156	Colônia Mato Grosso (Unir)	Colony	120	34	283.3
2015	399	Cujubim Grande	Ranch	228	34	149.1
2015	284	São Joao - Rio Madeira	Ranch	25	33	1320
2015	743	São Sebastião - Rio Madeira	Ranch	174	28	160.9
2015	709	Castanheira	Neighborhood	13014	27	2.1
2015	715	Bate Estaca	Neighborhood	1392	26	18.7
**2015**	**887**	**Bairro Novo (Br 364)**	**Neighborhood**	**1918**	**24**	**12.5**
2015	214	Linha H-27	Road	1350	24	17.8
2016	140	Km 14	Ranch	247	154	623.5
2016	121	Bacia Leiteira	Ranch	180	95	527.8
2016	737	Km13/Santa Helena	Farm	378	95	251.3
2016	337	Vista Alegre do Abunã	Village	3973	85	21.4
**2016**	**148**	**Colônia Viçosa**	**Colony**	**391**	**81**	**207.2**
2016	141	Uberaba	Ranch	167	70	419.2
2016	898	Vila do DNIT (8ª Região)	Village	4684	70	14.9
2016	152	Colônia Japonesa	Colony	98	68	693.9
2016	155	Areia Branca - Area Rural (Balneário)	Neighborhood	270	66	244.4
2016	151	Jerusalem da Amazonia	Ranch	120	50	416.7
2016	407	Maravilha (Rio Madeira)	Ranch	185	47	254.1
2016	320	Marmelo - Br 364 Pvh/Acre	Ranch	58	42	724.1
2016	739	São João	Ranch	126	42	333.3
**2016**	**396**	**Belmont**	**Ranch**	**585**	**38**	**65**
2016	22	Aeroclube	Neighborhood	2625	34	13
**2016**	**887**	**Bairro Novo (Br 364)**	**Neighborhood**	**1918**	**33**	**17.2**
2016	93	Estrada da Penitencia (P.A. Aliança)	Settlement	241	33	136.9
2016	35	Nacional	Neighborhood	8043	32	4
**2016**	**6**	**Nova Esperança**	**Neighborhood**	**11730**	**29**	**2.5**
2016	71	Mariana	Neighborhood	14055	28	2
2016	99	Terra Santa	Farm	328	25	76.2
2016	709	Castanheira	Neighborhood	13014	24	1.8
2016	53	Areia Branca	Neighborhood	1647	23	14
2016	145	São Joao do Garça	Ranch	51	23	451
2016	150	Vila Codaron - Km 13	Village	474	23	48.5
2016	86	Nova Porto Velho	Neighborhood	14748	21	1.4
2016	364	Bairro do Roque -Extrema	Neighborhood	539	20	37.1
2016	393	São Francisco - Rio Madeira	Ranch	35	20	571.4
2016	156	Colônia Mato Grosso (Unir)	Colony	120	19	158.3
2016	132	Km 08	Ranch	283	19	67.1
**2017**	**887**	**Bairro Novo (Br 364)**	**Neighborhood**	**1918**	**141**	**73.5**
2017	121	Bacia Leiteira	Ranch	180	111	616.7
2017	320	Marmelo - Br 364 Pvh/Acre	Ranch	58	89	1534.5
2017	737	Km13/Santa Helena	Farm	378	87	230.2
2017	337	Vista Alegre do Abunã	Village	3973	80	20.1
2017	140	Km 14	Ranch	247	78	315.8
2017	446	Nazaré (P.D.S. Nazaré E Boa Vitória)	Settlement	170	74	435.3
2017	443	Lago do Cuniã - Resex do Lago do Cuniã	Settlement	272	61	224.3
2017	151	Jerusalem da Amazonia	Ranch	120	60	500
2017	119	Uberaba	Ranch	240	60	250
2017	152	Colônia Japonesa	Colony	98	43	438.8
**2017**	**148**	**Colônia Viçosa**	**Colony**	**391**	**40**	**102.3**
2017	372	Nova California	Village	2650	39	14.7

2017	145	São Joao do Garça	Ranch	51	35	686.3
2017	110	Km 08	Ranch	260	30	115.4
2017	214	Linha H-27	Road	1350	30	22.2
2017	93	Estrada da Penitencia (P.A. Aliança)	Settlement	241	29	120.3
2017	317	Fortaleza do Abunã	Village	308	28	90.9
2017	898	Vila do DNIT (8ª Região)	Village	4684	28	6
**2017**	**396**	**Belmont**	**Ranch**	**585**	**27**	**46.2**
2017	407	Maravilha (Rio Madeira)	Ranch	185	26	140.5
**2017**	**6**	**Nova Esperança**	**Neighborhood**	**11730**	**26**	**2.2**
2017	213	Linha H-22	Road	149	25	167.8
2017	53	Areia Branca	Neighborhood	1647	24	14.6
2017	399	Cujubim Grande	Ranch	228	23	100.9
2017	841	Ramal Da Castanheira - (Pvh-Acre)	Road	112	23	205.4
2017	30	Aponiã	Neighborhood	18012	22	1.2
2017	709	Castanheira	Neighborhood	13014	22	1.7
2017	41	Cohab	Neighborhood	11589	22	1.9
2017	22	Aeroclube	Neighborhood	2625	19	7.2

Figure S1. Location of 25 sites sampled for anopheline presence in Porto Velho, Rondônia, Brazil. Four sites were selected: two urban (red dots) and two peri-urban sites (blue dots). Urban expansion area was depicted as the peripheral yellow area. Adapted from Google Earth (Image@2022 Maxar Technologies; Image@2022 CNES/Airbus) and from SEMPOG Google Maps (SEMPOG 2022).

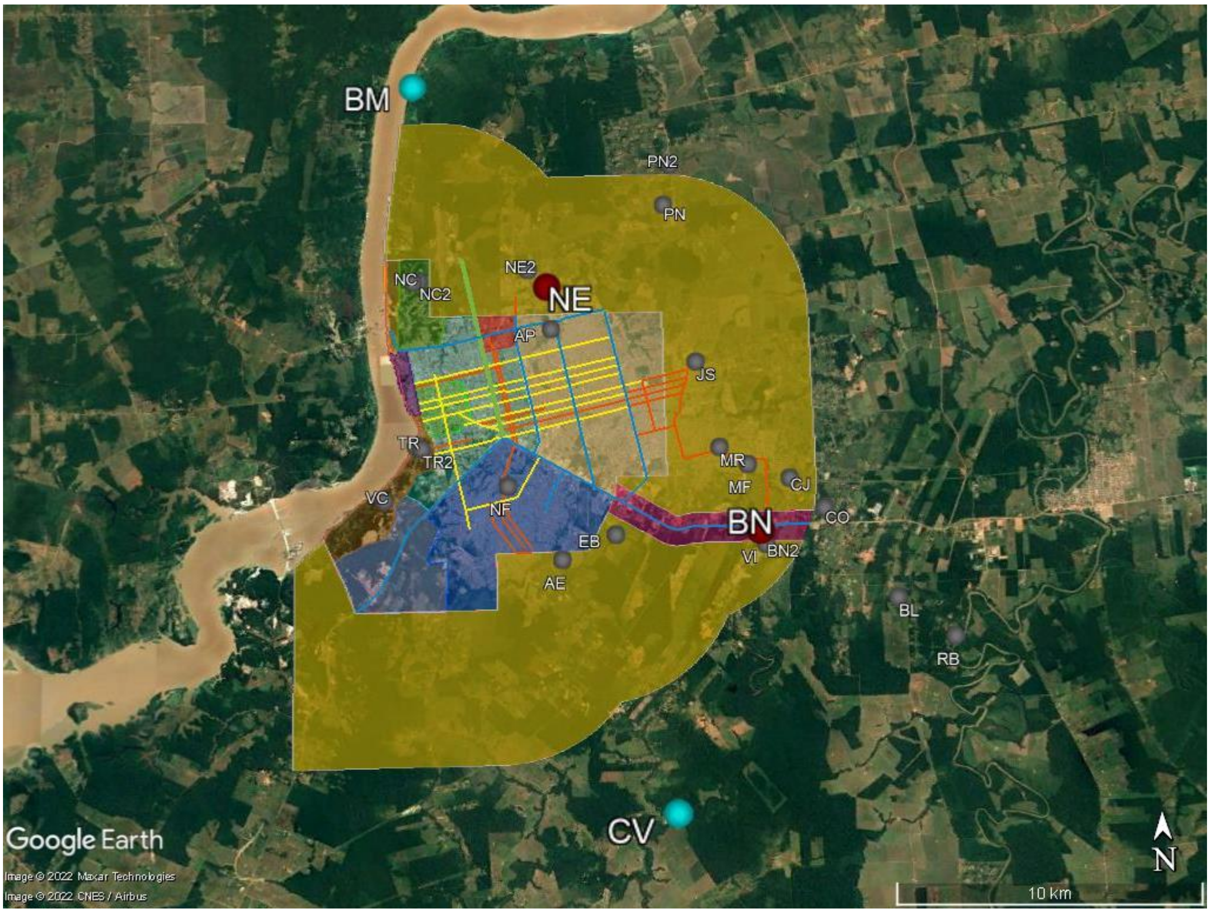

Table S2. Details on 25 sites where anopheline presence was recorded, and selected sites in bold.

Locality Name	Figure S1 Code	Locality Code SIVEP*	Settings	Latitude	Longitude	*Anopheles*** Occurrence
**Belmont**	**BM**	**396**	**Peri-urban**	**8°39'30.78"S**	**63°54'37.39"O**	**Positive**
Penal 1	PN	93	Peri-urban	8°41'57.50"S	63°49'50.90"O	+
Penal 2	PN2	93	Peri-urban	8°41'10.39"S	63°49'41.50"O	-
Bacia Leiteira	BL	121	Peri-urban	8°49'26.83"S	63°45'47.46"O	-
Embrapa	EB	-	Peri-urban	8°48'10.85"S	63°50'55.73"O	-
Ramal do Boto	RB	893	Peri-urban	8°50'9.84"S	63°44'46.88"O	+
Couve	CO	-	Peri-urban	8°47'47.12"S	63°47'3.34"O	+
Vila Candelária	VC	48	Peri-urban	8°47'27.21"S	63°55'11.84"O	-
**Colônia Viçosa**	**CV**	**148**	**Peri-urban**	**8°53'10.14"S**	**63°49'56.97"O**	**+**
**Nova Esperança 1**	**NE**	**6**	**Urban**	**8°43'31.46"S**	**63°52'6.12"O**	**+**
Nova Esperança 2	NE2	6	Urban	8°43'10.02"S	63°52'27.43"O	-
Triângulo 1	TR	44	Urban	8°46'27.23"S	63°54'36.37"O	+
Triângulo 2	TR2	44	Urban	8°46'30.39"S	63°54'29.87"O	-
Aeroclube	AE	22	Urban	8°48'36.62"S	63°51'55.50"O	+
Nacional 1	NC	35	Urban	8°43'21.03"S	63°54'38.43"O	-
Nacional 2	NC2	35	Urban	8°43'19.48"S	63°54'31.85"O	+
Mariana	MR	71	Urban	8°46'37.01"S	63°48'57.28"O	-
Marcos Freire	MF	70	Urban	8°46'56.86"S	63°48'26.58"O	-
Viena	VI	-	Urban	8°48'13.53"S	63°48'19.00"O	-
**Bairro Novo 1**	**BN**	**887**	**Urban**	**8°48'10.97"S**	**63°48'14.84"O**	**+**
Bairro Novo 2	BN2	887	Urban	8°48'25.31"S	63°48'10.00"O	-
Cidade Jardim	CJ	89	Urban	8°47'13.25"S	63°47'40.38"O	-
Nova Floresta	NF	56	Urban	8°47'14.38"S	63°52'54.20"O	-
Jardim Santana	JS	3	Urban	8°45'0.11"S	63°49'20.26"O	-
Aponiã	AP	30	Urban	8°44'18.75"S	63°52'2.64"O	-

* SIVEP MALARIA: Malaria Epidemiological Surveillance Information System

** *Anopheles* Occurrence: Presence (+) or absence (-) during one night-sampling.

Reference

SEMPOG - Municipal Planning, Budget and Management Department, PMPV - Prefeitura Municipal de Porto Velho (City Hall of Porto Velho). Institutional Planning and Strategic Management Department – DPGE. Available in: https://sempog.portovelho.ro.gov.br/artigo/19378/zoneamento. Access on April 18, 2022.
