# Supplementary material for: Anopheline diversity in urban and peri-urban malaria foci: comparison between alternative traps and seasonal effects in a city in the Western Brazilian Amazon
Source: Malar J. 2022 Sep 6;21:258. doi: 10.1186/s12936-022-04274-8 (PMC9450372; doi:10.1186/s12936-022-04274-8)
Supplement: Supplementary file 2 — Additional file 2. Residual Diagnostics, Model Validation, and Temporal Autocorrelation Analyses - Anopheline Abundance Data. [file 12936_2022_4274_MOESM2_ESM.pdf]

**Additional File 2**

Title of data: Residual Diagnostics, Model Validation, and Temporal Autocorrelation Analyses - Anopheline Abundance Data.

**Appendix S1**

Description of data: R codes used in the validation and selection of best-fitted models which estimates anopheline abundance according to predictors (Setting, Technique, Season) and random effects, sampling event, day (“DOY”), and sampling site. Due to the study design (consecutive three-day sampling), we performed an exploratory analysis to deal with temporal autocorrelation within each sampling event. More details on statistical approaches and packages can be accessed in Zuur et al. (2009), Brooks et al. (2017), Lüdecke (2018, 2021), and Hartig (2022).

```
##Model selection: BIC model comparisons and check for model assumptions (eg, residual adequacy: normality, homoscedasticity and outliers)
## R packages
library(ggplot2, sjPlot, sjmisc)
library(lme4, MASS)
library(DHARMa)
library(glmmTMB)
##Sampling event and day (DOY, day of year) were set as categorical variables
Sampling_event<-as.factor(Sampling_event)
DOY<-as.factor(DOY)
##GLMM (random and fixed effects) and GLM (fixed effects) were computed using negative
##binomial errors for abundance data and models ##with lowest BIC were selected and their
##residuals were visually inspected using DHARMa plots. The best-fitted model was highlighted in
##bold.
```

| Model | Formula - Abundance of *Anopheles* spp. | df | BIC |
|---|---|---|---|
| m1 | m1<-glmmTMB(Abundance~Setting+Technique+Season+(1\|Sampling_event/DOY)+(1\|Setting/Site), family=nbinom2(), data= Anopheles_Meireles_22) | 11 | 1187.741 |
| m2 | m2<-glmmTMB(Abundance~Setting+Technique+Season+(1\|DOY) + (1\|Site), family=nbinom2(), data= Anopheles_Meireles_22) | 9 | 1181.229 |
| m3 | m3<-glmmTMB(Abundance~Setting+Technique+Season+ (1\|Site), family=nbinom2(), data= Anopheles_Meireles_22) | 8 | 1214.571 |
| m4 | m4<-glmmTMB(Abundance~Setting+Technique+Season+(1\|DOY), family=nbinom2(), data= Anopheles_Meireles_22) | 8 | 1177.088 |
| **m5** | **m5<-glmmTMB(Abundance~Setting+Technique+Season+(1\|Sampling_event:DOY), family=nbinom2(), data= Anopheles_Meireles_22)** | **8** | **1177.088** |
| m6 | m6<-glm.nb(Abundance~Setting+Technique+Season, data= Anopheles_Meireles_22) | 7 | 1213.182 |
| a.null.model | a.nm<-glm.nb(Abundance~1, data= Anopheles_Meireles_22) | 2 | 1269.323 |

##Residual plots from the best-fitted model for the anopheline abundance estimates (model m5) using *DHARMa* package.
residuals.m5<- simulateResiduals(fittedModel = m5, plot=TRUE)

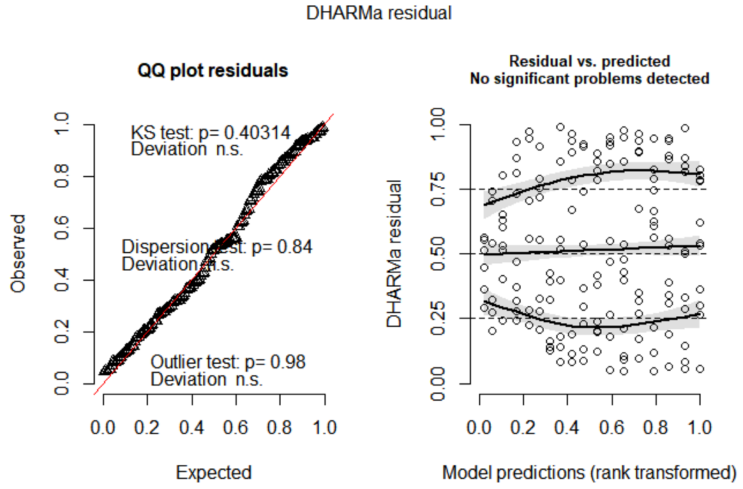


**##Complementar Analysis: Temporal Autocorrelation**
The responses obtained in a given sampling event may be related to our sampling design based on consecutive day sampling. In this way, we performed exploratory analysis to verify whether there was temporal autocorrelation within each sampling event, and its possible effect on anopheline abundance estimates. The best approximation model for anopheline abundance estimates (m5) already considers the temporal component as a random factor, thus preventing an undetected or unspecified autocorrelation. Model residuals were checked using testTemporalAutocorrelation() function from DHARMa package. No significative relationships were observed in the ACF plots or in the Durbin-Watson tests for abundance estimates; however, these findings must be interpreted with caution since the sample sizes were too small (days per sampling event) to detect any temporal trend.

**##model m5 – best-fitted model for anopheline abundance estimates – R code**
m5<-glmmTMB(Abundance~Setting+Technique+Season+ (1|Sampling_event:DOY),
family=nbinom2(), data= condensed_meireles_22)
res.m5<-simulateResiduals(fittedModel = m5)
**##temporal analysis – Abundance model m5 - R code. Temporal correlation structure was explored within each sampling event**
**##Six sampling events (months). Three-day consecutive sampling in each event.**
sampl.1.res.m5 = recalculateResiduals(res.m5, group =
condensed_meireles_22$Sampling_event==1)
testTemporalAutocorrelation(sampl.1.res.m5,
time=unique(condensed_meireles_22$Sampling_event==1))
sampl.2.res.m5 = recalculateResiduals(res.m5, group =
condensed_meireles_22$Sampling_event==2)
testTemporalAutocorrelation(sampl.2.res.m5,
time=unique(condensed_meireles_22$Sampling_event==2))
sampl.3.res.m5 = recalculateResiduals(res.m5, group =
condensed_meireles_22$Sampling_event==3)
testTemporalAutocorrelation(sampl.3.res.m5,
time=unique(condensed_meireles_22$Sampling_event==3))

```
sampl.4.res.m5 = recalculateResiduals(res.m5, group =
condensed_meireles_22$Sampling_event==4)
testTemporalAutocorrelation(sampl.4.res.m5,
time=unique(condensed_meireles_22$Sampling_event==4))
sampl.5.res.m5 = recalculateResiduals(res.m5, group =
condensed_meireles_22$Sampling_event==5)
testTemporalAutocorrelation(sampl.5.res.m5,
time=unique(condensed_meireles_22$Sampling_event==5))
sampl.6.res.m5 = recalculateResiduals(res.m5, group =
condensed_meireles_22$Sampling_event==6)
testTemporalAutocorrelation(sampl.6.res.m5,
time=unique(condensed_meireles_22$Sampling_event==6))
```

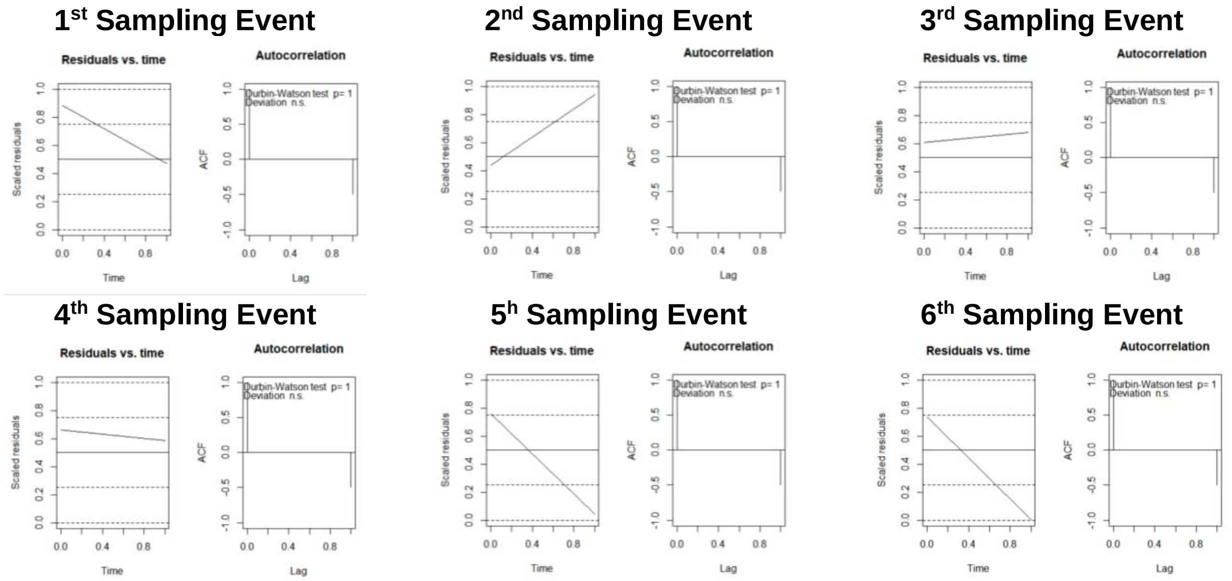


**References**

Brooks ME, Kristensen K, Van Benthem KJ, Magnusson A, Berg CW, Nielsen A, Skaug HJ, Mächler M, Bolker B M. glmmTMB balances speed and flexibility among packages for zero-inflated generalized linear mixed modeling. The R journal, 2017, 9(2):378-400.

Hartig F. DHARMa: residual diagnostics for hierarchical (multi-level/mixed) regression models. 2022-01-16. Available in: https://cran.rproject.org/web/packages/DHARMa/vignettes/DHARMa.html. Access on May 11, 2022.

Lüdecke D. Statistics for Mixed Effects Models. 2018-02-04 Available in: http://cran.nexr.com/web/packages/sjstats/vignettes/mixedmodels-statistics.html. Access on May 11, 2022.

Lüdecke D. Summary of Regression Models as HTML Table. 2021-11-26. Available in: https://cran.r-project.org/web/packages/sjPlot/vignettes/tab_model_estimates.html. Access on May 11, 2022.

Zuur AF, Ieno EN, Walker NJ, Saveliev AA, Smith GM Mixed effects models and extensions in ecology with R (Vol. 574). 2009, New York: Springer.
