# Supplementary material for: Anopheline diversity in urban and peri-urban malaria foci: comparison between alternative traps and seasonal effects in a city in the Western Brazilian Amazon
Source: Malar J. 2022 Sep 6;21:258. doi: 10.1186/s12936-022-04274-8 (PMC9450372; doi:10.1186/s12936-022-04274-8)
Supplement: Supplementary file 3 — Additional file 3. Non-anopheline mosquitoes and other Dipterans. Table S3. Number of non-anopheline mosquitoes, biting midges, and sand flies caught with Gazetrap, PHLC, and Screen, at urban and peri-urban settings. Table S4. Number of non-anopheline mosquitoes, biting midges, and sand flies caught during seasonal periods, at urban and peri-urban settings. [file 12936_2022_4274_MOESM3_ESM.pdf]

Additional File 3

Title of data: Non-anopheline mosquitoes and other Dipterans

Description of data: Table with the number of non-anopheline mosquitoes, biting midges (Ceratopogonidae: *Culicoides* spp.), and sand flies (Psychodidae: Phlebotominae) caught with Gazetrap, PHLC, and Screen (Table S3), over three seasonal periods (Table S4), in the urban and peri-urban settings of Porto Velho, state of Rondônia, Brazilian Amazon.

Table S3. Number of non-anopheline mosquitoes, biting midges, and sand flies caught with Gazetrap, PHLC, and Screen, at urban and peri-urban settings.

Taxa	Urban Setting			Peri-urban Setting		
	Gazetrap	PHLC	Screen	Gazetrap	PHLC	Screen
Culex	292	557	170	309	184	139
Mansonia	47	27	7	398	434	127
Coquillettidia	1	-	4	87	70	5
Aedes	44	62	12	4	7	2
Aedeomyia	25	2	2	8	4	15
Psorophora	-	-	-	4	-	2
Uranotaenia	-	-	1	2	-	-
Wyeomyia	-	-	-	3	-	-
Ceratopogonidae: *Culicoides* spp.	35	30	-	28	3	-
Psychodidae: Phlebotominae	1	-	6	11	2	1
Total	445	678	202	854	704	291

Table S4. Number of non-anopheline mosquitoes, biting midges, and sand flies caught during seasonal periods, at urban and peri-urban settings.

Taxa	Urban Setting			Peri-urban Setting		
	Transition	Dry	Rainy	Transition	Dry	Rainy
Culex	378	337	304	91	90	451
Mansonia	7	63	11	494	345	120
Coquillettidia	1	4	-	98	37	27
Aedes	44	3	71	8	1	4
Aedeomyia	22	6	1	1	24	2
Psorophora	-	-	-	2	1	3
Uranotaenia	1	-	-	1	1	-
Wyeomyia	-	-	-	-	1	2
Ceratopogonidae: *Culicoides* spp.	24	11	30	21	2	8
Psychodidae: Phlebotominae	3	1	3	8	2	4
Total	480	425	420	724	504	621
