# Supplementary material for: Anopheline diversity in urban and peri-urban malaria foci: comparison between alternative traps and seasonal effects in a city in the Western Brazilian Amazon
Source: Malar J. 2022 Sep 6;21:258. doi: 10.1186/s12936-022-04274-8 (PMC9450372; doi:10.1186/s12936-022-04274-8)
Supplement: Supplementary file 4 — Additional file 4. Supplementary results for the number of individuals/species of Anopheles. Model Output – Best-fitted models. PERMANOVA / PERMDISP estimates and NMDS plots. Table S5. Anopheles species and individuals (N) captured at urban and peri-urban settings of Porto Velho, state of Rondônia, Brazilian Amazon. Table S6. Anopheles species and individuals (N) captured through Gazetrap, PHLC, and Barrier Screen in Porto Velho, state of Rondônia, Brazilian Amazon. Table S7. Anopheles species and individuals (N) captured in the Dry, Rainy, and Transitional seasons, at Porto Velho, state of Rondônia, Brazilian Amazon. Table S8. Summary of estimates from the best approximation models for anopheline abundance and richness. Table S9. PERMANOVA and PERMDISP analyses for Anopheles assemblage structure between seasons and sampling techniques. Fig. S2. Non-metric multidimensional scaling (NMDS) plots for graphical visualization of the PERMANOVA and PERMDISP analyses of Anopheles assemblage structure. [file 12936_2022_4274_MOESM4_ESM.pdf]

Additional File 4

Title of data: Supplementary results for the number of individuals/species of *Anopheles*.

Description of data: Tables with the *Anopheles* species and individuals (N) captured in urban and peri-urban settings, using Gazetrap, PHLC, and Barrier Screen techniques, during the Dry, Rainy, and Transitional seasons. Collections were done at the city of Porto Velho, state of Rondônia, Brazilian Amazon.

Table S5. *Anopheles* species and individuals (N) captured at urban and peri-urban settings of Porto Velho, state of Rondônia, Brazilian Amazon.

Species	Urban		Peri-urban		Total
	Indiv.	%	Indiv.	%	
An. darlingi	284	54.51	2,279	93.36	2,563
An. triannulatus	176	33.78	1	0.04	177
An. mattogrossensis	2	0.38	94	3.85	96
An. konderi	1	0.19	43	1.76	44
An. strodei	17	3.26	1	0.04	18
An. nuneztovari	9	1.73	8	0.33	17
Anopheles spp.	9	1.73	8	0.33	17
An. benarrochi	10	1.92	-	-	10
An. deaneorum	2	0.38	4	0.16	6
An. argyritarsis	4	0.77	1	0.04	5
An. minor	3	0.58	1	0.04	4
An. mediopunctatus/costai/forattini	2	0.38	-	-	2
An. peryassui	1	0.19	1	0.04	2
An. braziliensis	1	0.19	-	0.00	1
Total	521	17.59	2441	82.41	2,962

Table S6. *Anopheles* species and individuals (N) captured through Gazetrap, PHLC, and Barrier Screen in Porto Velho, state of Rondônia, Brazilian Amazon.

Species	Gazetrap		PHLC		Screen		Total
	N	%	N	%	N	%	
An. darlingi	535	20.87	1473	57.47	555	21.65	2,563
An. triannulatus	39	22.03	106	59.89	32	18.08	177

An. mattogrossensis	56	58.33	18	18.75	22	22.92	96
An. konderi	7	15.91	22	50	15	34.09	44
An. strodei	2	11.11	11	61.11	5	27.78	18
An. nuneztovari	5	29.41	8	47.06	4	23.53	17
Anopheles spp.	2	11.76	12	70.59	3	17.65	17
An. benarrochi	3	30	7	70	-	-	10
An. deaneorum	3	50	2	33.33	1	16.67	6
An. argyritarsis	2	40	2	40	1	20	5
An. minor	3	75	-	-	1	25	4
An. mediopunctatus/costai/forattini	-	-	-		2	100	2
An. peryassui	1	50	1	50	-	-	2
An. braziliensis	1	100	-	-	-	-	1
Total	659	22.25	1662	56.11	641	21.64	2,962

Table S7. *Anopheles* species and individuals (N) captured in the Dry, Rainy, and Transitional seasons, at Porto Velho, state of Rondônia, Brazilian Amazon.

Species	Dry		Rainy		Transition		Total
	N	%	N	%	N	%	
An. darlingi	1145	44.67	691	26.96	727	28.37	2,563
An. triannulatus	95	53.67	25	14.12	57	32.20	177
An. mattogrossensis	14	14.58	13	13.54	69	71.88	96
An. konderi	2	4.55	22	5	20	45.45	44
An. strodei	14	77.78	-	-	4	22.22	18
An. nuneztovari	4	23.53	8	47.06	5	29.41	17
Anopheles spp.	4	23.53	6	35.29	7	41.18	17
An. benarrochi	-	-	3	30	7	70	10
An. deaneorum	1	16.67	-	-	5	83.33	6
An. argyritarsis	3	60	1	20	1	20	5
An. minor	2	50	-	-	2	50	4
An. mediopunctatus/costai/forattini	2	100	-	-	-	-	2
An. peryassui	-	-	-	-	2	100	2
An. braziliensis	1	100	-	-	-	-	1
Total	1287	43.45	769	25.96	906	30.59	2,962

Title of data: Model Output – Best-fitted models

Description of data: Generalized linear mixed model (GLMM) with negative binomial errors for the effects of city setting, sampling technique, season, and random effects on *Anopheles* spp. abundance. Summary output from the null model GLM examining anopheline richness

estimates (Poisson errors). All estimates were transformed in the *tab_model* command from *sjplot* package (see Lüdecke 2021).

Table S8. Summary of estimates from the best approximation models for anopheline abundance and richness.

Predictors	**Abundance – GLMM**			
	*IRR**	*CI*	*z value*	*p*
Fixed effects				
Intercept**	16.24	9.10 – 28.99	9.43	**<0.001**
Urban Setting	0.28	0.21 – 0.38	-8.61	**<0.001**
PHLC Technique	2.35	1.74 – 3.16	5.61	**<0.001**
Screen Technique	0.90	0.64 – 1.25	-0.65	0.514
Rainy Season	0.63	0.30 – 1.35	-1.18	0.238
Transition Season	0.67	0.31 – 1.42	-1.05	0.292
Random effects				
σ^2 = 0.45	Within-group-variance			
τ_{00} = 0.70 $_{Sampling\_event:DOY}$	Between-group-variance			
ICC = 0.61	Between-group-variance/Total variance			
N 6 $_{Sampling\_event}$				
36 $_{DOY}$				
Observations	162			
Marginal R^2 ***	0.031			
Conditional R^2***	0.067			
	**Richness - Null Model - GLM**			
	*IRR**	*CI*	*z value*	*p*
Intercept	1.19	0.99 – 1.43	1.85	0.064

Legend: *Incidence Rate Ratios **Intercept: The basal levels of all predictors (Peri-urban Setting, Gazebo Technique, Dry Season). *p* = Pseudo R-squared statistics; *CI* = Confidence intervals. Random effects: σ^2 = Residual variance of random effects; τ_{00} = Tau-00, Variation between individual intercepts and average intercept; ICC = Intraclass-Correlation Coefficient; Marginal R^2 = variance explained by fixed effects; Conditional R^2 = variance explained by fixed and random effects. R^2*** = Pseudo R-squared statistics proposed by Nakagawa et al. (2017).

Notes on Marginal R^2 and Conditional R^2

Marginal (R^2m) and Conditional (R^2c) r-squared were computed using MuMIn R package (Bartoń 2020) which proposes delta method, lognormal approximation, and trigamma function

for deriving variance. The trigamma-estimates were recommended whenever available for model family/structure, and according to Nakagawa et al. (2017), trigamma function provides the most accurate estimate of the observation-level variances.

```
library(MuMIn)          # R code for the package
r.squaredGLMM(m5)    # R code for function
```

Method	R^2m	R^2c
delta	0.11515869	0.24202203
lognormal	0.21283918	0.44731120
trigamma	0.03185788	0.06695378

Title of data: PERMANOVA/PERMDISP estimates and NMDS plots

Description of data: There were no significant patterns in the *Anopheles* assemblage structure when seasons and sampling techniques were compared by Permutational Multivariate Analysis of Variance (PERMANOVA) and Permutational Analysis of Multivariate Dispersions (PERMDISP) tests. Nonmetric multidimensional scaling (NMDS) plots were used to depict *Anopheles* assemblage dissimilarities among predictor levels. The R *vegan*, *lattice*, and *permute* packages were used in the compositional analyses.

Table S9. PERMANOVA and PERMDISP analyses for *Anopheles* assemblage structure between seasons and sampling techniques.

Permutational Analysis	Source	df	SS	MS	pseudo-F	R^2	P
	Season	2	0.265	0.132	1.114	0.129	0.382
Multivariate	Residuals	15	1.787	0.119		0.870	
Variance	Total	17	2.053			1.000	
(PERMANOVA)	Technique	2	0.080	0.040	0.173	0.037	0.985
	Residuals	9	2.093	0.232		0.962	
	Total	11	2.174			1.00	
Multivariate	Season	2	0.064	0.032	1.230		0.319
beta-dispersion	Residuals	15	0.390	0.026			
(PERMDISP)	Technique	2	0.011	0.005	0.166		0.834
	Residuals	9	0.307	0.034			

PERMANOVA and PERMDISP were based on Sørensen dissimilarities. Abbreviations: df = degrees of freedom, SS = sum of squares, MS = mean sum of squares, pseudo-F = F value obtained by 999 permutations. Season levels: Dry, Rainy, and Transitional seasons. Technique levels: Gazetrap, PHLC, and Screen.

Fig. S2. Non-metric multidimensional scaling (NMDS) plots for graphical visualization of the PERMANOVA and PERMDISP analyses of *Anopheles* assemblage structure. **a** Assemblage distances between Dry, Rainy, and Transition seasons (Stress=0.137). **b** Assemblage distances between Gazetrap, PHLC, and Screen techniques (Stress= 0.061). Compositional distances were computed through matrices of Sørensen dissimilarities. See PERMANOVA and PERMDISP results in Table S9.

a **b**

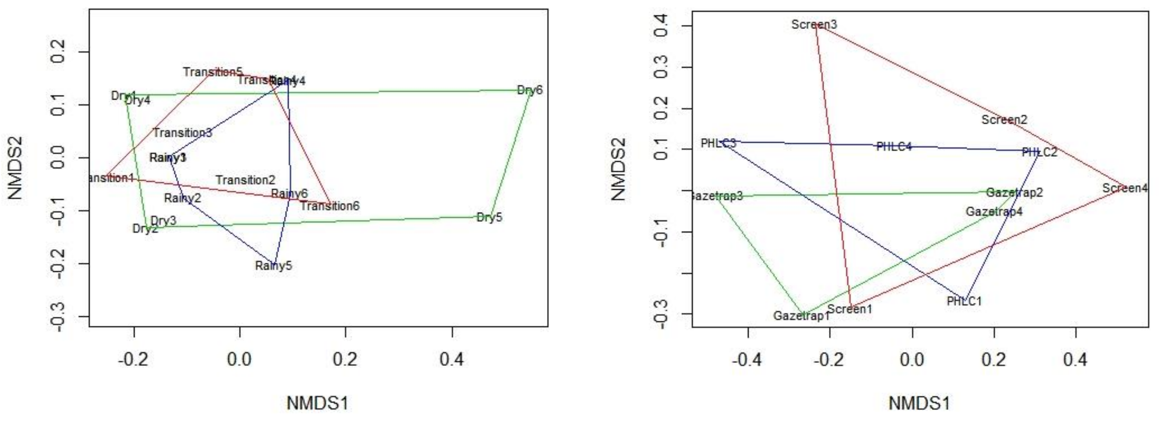

References

BARTOŃ, K. MuMIn: multi-model inference. R package version 1.43. 17. Retrieved May, v. 11, p. 2021, 2020.

Lüdecke D. Summary of Regression Models as HTML Table. 2021-11-26. Available in: https://cran.r-project.org/web/packages/sjPlot/vignettes/tab_model_estimates.html. Access on May 11, 2022.

Nakagawa S, Johnson PC, Schielzeth H. The coefficient of determination R^2 and intra-class correlation coefficient from generalized linear mixed-effects models revisited and expanded. J R Soc. Interface, 2017; 14(134):20170213. doi: 10.1098/rsif.2017.0213
